# Phenomic and Genomic Characterization of a Mutant Platform in *Cucurbita pepo*

**DOI:** 10.3389/fpls.2018.01049

**Published:** 2018-08-03

**Authors:** Alicia García, Encarni Aguado, Genis Parra, Susana Manzano, Cecilia Martínez, Zoraida Megías, Gustavo Cebrián, Jonathan Romero, Sergi Beltrán, Dolores Garrido, Manuel Jamilena

**Affiliations:** ^1^Department of Biology and Geology, Research Centers CIAIMBITAL and CeiA3, University of Almería, Almería, Spain; ^2^Centro Nacional de Análisis Genómico, Barcelona, Spain; ^3^Departamento de Fisiología Vegetal, Facultad de Ciencias, Universidad de Granada, Granada, Spain

**Keywords:** *Cucurbita pepo*, EMS, mutant platform, WGS, high-throughput screening, ethylene

## Abstract

The *Cucurbita pepo* genome comprises 263 Mb and 34,240 gene models organized in 20 different chromosomes. To improve our understanding of gene function we have generated an EMS mutant platform, consisting of 3,751 independent M2 families. The quality of the collection has been evaluated based on phenotyping and whole-genome re-sequencing (WGS) results. The phenotypic evaluation of the whole platform at seedling stage has demonstrated that the rate of variation for easily observable traits is more than 10%. The percentage of families with albino or chlorotic seedlings exceeded 3%, similar or higher to that found in other EMS collections of cucurbit crops. A rapid screening of the library for triple ethylene response in etiolated seedlings allowed the identification of four ethylene-insensitive mutants, that were found to be semidominant (*ein1*, *ein2*, and *ein3*) or dominant (*EIN4*). By evaluating 4 adult plants from 300 independent families more than 28% of apparent mutations were found for vegetative and reproductive traits, including plant vigor, leaf size and shape, sex expression and sex determination, and fruit set and development. Two pools of genomic DNA derived from 20 plants of two mutant families were subjected to WGS by using NGS methodology, estimating the density, spectrum, distribution and impact of EMS induced mutation. The number of EMS mutations in the genomes of families L1 and L2 was 1,704 and 859, respectively, which represents a density of 11.8 and 6 mutations per Mb, respectively. As expected, the predominant EMS induced mutations were C > T and G > A transitions (80.3% in L1, and 61% L2), that were found to be randomly distributed along the 20 chromosomes of *C. pepo*. The mutations were mostly affecting intergenic regions, but 7.9 and 6% of the identified EMS mutations in L1 and L2, respectively, were located in the exome, and 0.4 and 0.2% had a moderate and high putative impact on gene functions. These results provide information regarding the potential use of the obtained mutant platform in the discovery of novel alleles for both functional genomics and *Cucurbita* breeding by using direct- or reverse-genetic approaches.

## Introduction

The genus *Cucurbita* comprises three important worldwide-cultivated crop species: *Cucurbita pepo, C. maxima*, and *C. moschata*. The most important cultivated species of the genus is *C. pepo*, consisting of different morphotypes of summer and winter squashes and gourds with great economic importance such as Zucchini, Pumpkin, Vegetable Marrow, Cocozelle, Scallop, Acorn, Straightneck and Crookneck. In the year 2014 the cultivated area of *C. pepo* reached nearly 2 million of hectares and a production of 25 million of tons (FAOSTAT, 1961).

The genome of *C. pepo* has been recently sequenced and annotated^1^ (Montero-Pau et al., 2017). It consists of 263 Mb, a scaffold N50 of 1.8 Mb, and 34,240 gene models, organized in 20 chromosomes that cover about 93% of the assembled sequence. A duplication event has been detected in the genome of *Cucurbita*, happening about 20 Mya ago, after the separation from other genus of the *Cucurbitaceae* family such as *Citrullus* (watermelon) and *Cucumis* (melon and cucumber) (Montero-Pau et al., 2017; Sun et al., 2017).

Although the drafts of the genomes of *C. pepo* and other cucurbit species are becoming more complete, little is known about specific gene functions in these crops. Chemically induced plant mutant platforms, such as those generated by EMS, a chemical mutagen that induces single randomly distributed nucleotide changes in DNA (Sega, 1984; Greene et al., 2003), have become an important source of variability for both functional genomic analyses and for plant breeding programs. Once established, the mutant platforms can be used for direct phenotyping, but also for DNA screenings, allowing the detection of single point mutations in a number of specific genes.

High-throughput screenings based on plant phenotyping of large mutant collections are becoming increasingly important as a source of new useful mutants in crop species. The difficulty of direct screenings lies in the large number of plants to be evaluated for each of the characters under study. Since mutations are mostly recessive, the identification of a mutant phenotype requires 8–10 plants to be phenotyped per family, and therefore the screening of 1,000 mutant families requires the assessment 8,000–10,000 plants. Massive screenings based on seedling early traits have been developed for the detection of agronomic interesting mutants, including mutants with tolerance to biotic and abiotic stresses, as well as insensitive mutants to certain plant hormones or to chemical treatments with specific herbicides or pesticides. For example, the molecular bases of ethylene-regulated processes have been discerned thanks to the analysis of *Arabidopsis* mutants which are altered in the seedling triple response to ethylene (Bleecker et al., 1988). The response of seedlings to ethylene have been successfully used to screen different EMS mutant collections in *Arabidopsis* (Guzmán and Ecker, 1990), which allowed the identification of a number of mutants altered in the ethylene pathway (Roman et al., 1995).

Indirect screenings were become more popular since the development of the TILLING (Targeting Induced Local Lesions in Genomes) reverse genetic approach (Colbert et al., 2001). This methodology was firstly applied to detect allelic variants of an specific gene in *Arabidopsis* EMS mutant collections (Greene et al., 2003), but its utilization was rapidly extended to a number of crop species, including rice (Till et al., 2007), maize (Till et al., 2004), barley (Caldwell et al., 2004), *Brassica napus* (Wang et al., 2008), tomato (Minoia et al., 2010), among others. In cucurbit species, TILLING platforms were developed, allowing the detection of new allelic variants for plant breeding in *C. melo* (Dahmani-Mardas et al., 2010; González et al., 2011) and *C. sativus* (Boualem et al., 2014; Fraenkel et al., 2014). An EMS mutant collection has already been published in *C. pepo*, and different target genes were assessed by TILLING (Vicente-Dólera et al., 2014), but the number of mutant squash families is still very poor.

In this paper we present the development and utilization of a new EMS mutant platform in *C. pepo*, made of 3,751 M2 families. The quality and utility of the collection was confirmed by phenomic screenings of the mutant plants at both seedling and adult stage of development, by using a forward high-throughput screening for ethylene insensitivity, and by assessing the mutation rate and distribution of mutations in two families by whole genome re-sequencing. Results demonstrated that the collection has sufficient quality to address the identification of new *C. pepo* mutants throughout forward genetics, as well as the high density and random distribution of induced mutations in the genome of this crop species for reverse genetics. The established mutant platform constitutes therefore an important resource for functional genomic studies, but also a source of new alleles for squash breeding programs.

## Materials and Methods

### Generation of the EMS Mutant Platform

The mutant library was generated in an inbred line derived from the Spanish landrace MUC16. Before starting the generation of the EMS mutant collection, we established the optimal EMS concentration producing the higher mutation density without much altering the seed germination rate, neither the fertility of the M1 plants. For this end, batches of 200 mature seeds of MUC16 were immersed in bottles containing 0–2% EMS in 200 ml of deionized water and stirred in a shaker for either 12 or 24 h at 22°C in darkness. After the EMS treatment, seeds were washed twice in 200 ml of 3% Na_2_S_2_O_3_ buffer for 30 min at room temperature with gentle shaking, followed by three washes in 200 ml distilled water for 30 min each. Control seeds were treated with distilled water in the same manner. Treated seeds were germinated in a commercial nursery following standard local practices. After evaluating the germination rate, seedlings were grown under standard greenhouse conditions in Almería (Spain) to obtain mature M1 plants. Each M1 plant was then self-pollinated, and M2 seeds extracted from individual fruits and stored. The fertility of M1 plants was assessed as the percentage of plants producing more than 20 viable seeds per fruit, i.e., those fruits producing less than 20 seeds were considered to be derived from male- or female-sterile plants. From those experiments we concluded that the optimal mutagenic treatment was that containing 0.3% EMS for a total of 12 h.

**FIGURE 1:**
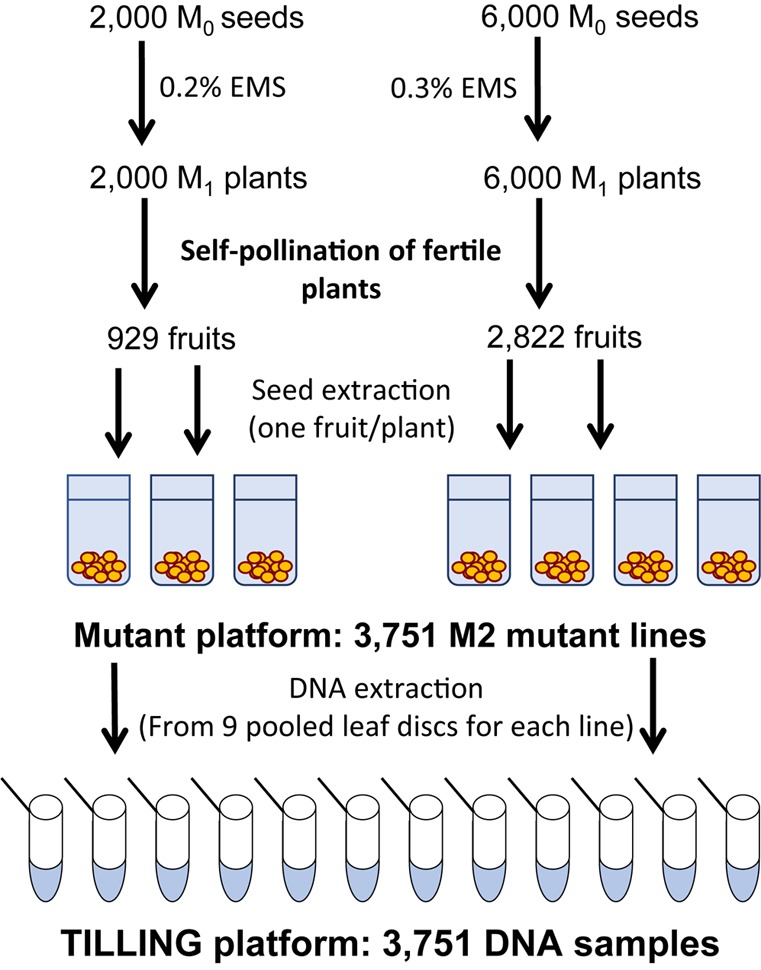
Steps in the construction of the mutant and TILLING platforms of *C. pepo*, each one composed of 3,751 seed and DNA samples, respectively.

To generate the *C. pepo* mutant collection, we treated 6,000 and 2,000 MUC16 seeds with either 0.3 or 0.2% EMS for 12 h, respectively. Those EMS doses did not reduce the germination rate of the MUC16 seeds but lessened the fertility of the plants to nearly 50%. Plants derived from the 6,000 and 2,000 treated seeds were selfed to achieve a total of 2,822 and 929 fruits with more than 20 viable seeds (**Figure 1**). Consequently, the generated mutant collection comprises a total of 3,751 M2 families. Regarding the mutant seeds availability, we currently have an average of 160 seeds per M2 family, and around 600 seeds for each of the 900 M3 families that were already propagated.

### Screening of the Mutant Library and Generation of a TILLING Platform

The quality of the mutant collection was firstly tested by evaluating the percentage of easily observable traits in 9 seedlings of each mutant family. A total of 30,008 seedlings were grown, and the alterations in the development and pigmentation of cotyledons and first leaves, in comparison with the background genotype MUC16, recorded for each plant. For each M2 mutant family we also collected together one leaf disk from eight individual seedlings for DNA extraction, developing so a TILLING platform consisting of 3,751 DNA samples.

The M2 plants from 284 families in the 0.3% treatment were also grown to maturity under standard greenhouse conditions, determining the percentage of alterations in vegetative and reproductive developmental traits, including plant vigor and height, leaf morphology, root conformation, female and male flower development, sex related traits, and fruit color, size, shape. These M2 families were also selfed to obtain the M3 generation.

### Assay of Triple Response to Ethylene

To test the quality of the squash mutant collection we also performed a high-throughput screening of the 3,751 M2 families for mutants in the ethylene-response pathway. The triple response of etiolated seedlings to ethylene was so used to detect M2 families segregating for Ein mutants. Eight seeds of each M2 family were sown in seedlings trays, germinated for 2 days in the absence of ethylene and then introduced into a growth chamber containing 50 ppm of ethylene in darkness for a total of 5 days. The Ein seedlings were perfectly distinguishable since they did not reduce the length of their hypocotyls, protruding therefore over the rest of the ethylene-sensitive seedlings in the growth chamber. By using this ethylene test we detected four segregating M2 families. The ethylene insensitivity of these four families was confirmed by evaluating the response to ethylene within a higher number of plants in each selected M2 family. The Ein plants were then backcrossed with the inbred line MUC16 for two generations and the resulting progeny (BC_2_) selfed to achieve the BC_2_S_1_ generation. These generation was again evaluated by the ethylene triple response.

Data were analyzed by multiple comparisons by analysis of variance (ANOVA) with significance level *p* < 0.05, and each two means were compared with the Fisher’s least significant difference (LSD) method. The chi-square analyses were performed using the statistical software Statgraphics Centurion XVI.

### Library Preparation and Sequencing

Whole genome sequencing was performed at the Centro Nacional de Análisis Genómico (CNAG, Barcelona, Spain). Four DNA samples were sequenced for two random M_2_ families from the *C. pepo* mutant collection: family 435 corresponding to mutant family 1 (L1), and family 1717 corresponding to mutant family 2 (L2). Each sample comprises 10 random plants from each family. Paired-end multiplex libraries were prepared according to manufacturer’s instructions with KAPA Library Preparation kit (Kapa Biosystems). Libraries were loaded to Illumina flowcells for cluster generation prior to producing 126 base read pairs on a HiSeq2000 instrument following the Illumina protocol. Base calling and quality control was done with the Illumina RTA sequence analysis pipeline according to manufacturer’s instructions. Based on the genomic sequence of *Cucurbita pepo* (genome v4.1) the average fold effective coverage of all the samples was in the range between 13.18 and 17.90 (Supplementary Table 1).

### Data Analysis

Sequencing reads were trimmed to avoid any remaining adaptors. Resulting reads were mapped to the *C. pepo* genome v4.1^2^ using the GEM3 toolkit (Marco-Sola et al., 2012) allowing up to 8% mismatches. Alignment files (BAM format) containing only properly paired, uniquely mapping, reads were processed using picard tools version 1.110 (Picard Tools - By Broad Institute) to add read groups and remove duplicates. The Genome Analysis Tool Kit (GATK) (McKenna et al., 2010) version 3.6 was used for local realignment and base recalibration. Processed BAM files were submitted to variant calling for SNVs and small insertions and deletions using HaplotypeCaller (GATK v3.6). Functional annotations were added to the resulting VCF using SnpEff (Cingolani et al., 2012) with the gene annotation provided for *C. pepo* genome v4.1^2^.

### Variant Filtering

Variants were removed when depth of coverage was less than 8 or greater than 100 in at least one of the samples of each family (L1 and L2). This allowed us not only to exclude positions for which the coverage depth was low, but also positions that might fall in segmental duplications. From the whole genome (260 Mb), after filtering we keep 174 Mb for family L1 and 178 Mb for family L2.

Wrongly mapped reads are difficult to account for and can result in an increased false discovery of genetic variants. In order to remove undetected duplications or repetitive regions that could mislead the alignment program, we further filtered out genomic regions with unusually high number of variants. To identify those regions, a sliding window approach was used, with a window size of 1 kb and a step size of 500 bp. All genomic 1 kb windows with more than one variant where discarded. As a result, we discarded 25 Mb of total genomic regions from L1 and 26 Mb from L2.

To determine if a mutation has been caused by EMS in one of the families, it is necessary to have reliable information for that particular position in both families (L1 and L2). After applying the coverage filter removing the hyperpolymorphic regions the intersection of the L1 and L2 genomic positions was computed. The shared reliable regions contained a total of 144.5 Mb.

## Results

### Generation of an EMS Mutant Collection in *C. pepo*

**FIGURE 2:**
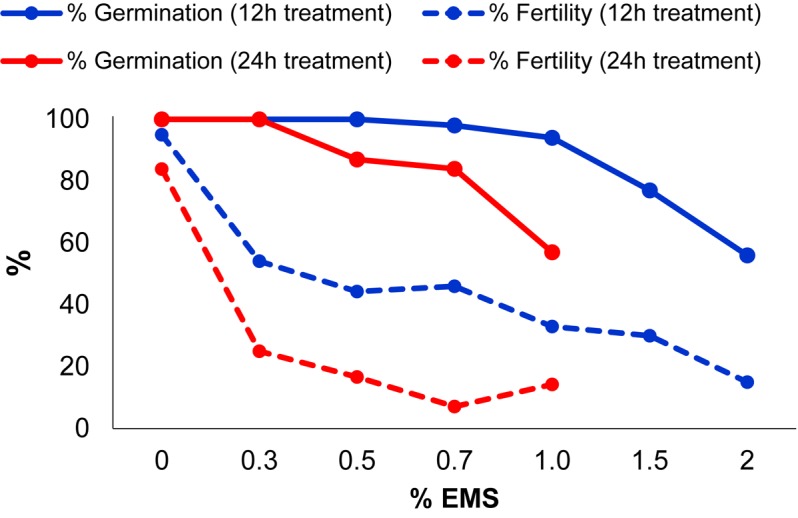
Effect of different EMS treatments on M1 seed germination and plant fertility.

In order to optimize the EMS induced mutagenesis, batches of 200 seeds of MUC16, a *C. pepo* inbred line that was also used for genome sequencing (Montero-Pau et al., 2017), were treated with different concentrations of EMS for 12 and 24 h. The efficiency of the mutagenesis treatments was then estimated by assessing two parameters on M1 plants: seed germination and plant fertility. **Figure 2** shows the reduction in the percentage of germination and in the fertility of M1 plants subjected to the different treatments.

In seeds treated for 12 h, no significant reduction in germination rates was observed in 0.3–1% EMS treatments, but in 1.5 and 2% EMS treatments the germination was highly reduced (**Figure 2**). Most of the M1 plants which were treated for 12 h, exhibited growth retardation at seedling stages, but all of them recovered and flowered. We found an inverse correlation between mutagen dose and M1 plant fertility, assessed as the number of selfed fruits, each one having more than 20 viable seeds (**Figure 2**). At 1.5 and 2% EMS, 70 and 85% of the plants were considered sterile because they either did not set fruit or yielded fruits with less than 20 seeds after selfing.

In EMS treatments for 24 h, the 1.5 and 2% treatment were omitted. Even so, the germination rate was highly reduced, reaching 57% germination after 1% EMS treatment (**Figure 2**). The fertility of the M1 plants was greatly reduced in EMS treatments for 24 h, showing less than 75% fertility in the treatments with the different doses (**Figure 2**). Taken together, the results demonstrated that, even at levels that barely reduced the germination rate, the fertility of plants was strongly affected. Therefore, the most appropriated EMS dosage to produce the mutant collection was selected based on M1 plant fertility. The 0.3% EMS for 12 h was selected as the most suitable treatment for mutagenesis of MUC16 seeds. This EMS dosage did not affect the germination rate of the M1 plants, but reduced M1 plant fertility to about 54% (**Figure 2**). Fertile plants yielded fruits that contained more than 100 seeds after selfing, therefore not compromising the production of the M2 generation. In addition, we selected a slightly reduced EMS concentration (0.2%) to obtain one third of the mutant families in our collection.

For generating the final mutant collection, 8,000 MUC16 seeds were mutagenized; 6.000 were treated with 0.3% EMS and 2000 seeds with 0.2% EMS for 12 h at 22°C. The germinated seed was grown in a greenhouse and each M1 fertile plant selfed to obtain the M2 offspring. A total of 3,751 M2 families were obtained, 929 and 2,822 for the 0.2 and 0.3% doses of EMS, respectively (**Figure 1**). Nine plants were grown from each mutagenized family, and leaf material from each plant was collected and mixed for DNA extraction. A total of 3,751 samples of DNA were isolated to establish a TILLING platform of the *C. pepo* mutant collection (**Figure 1**).

### Phenotypic Variations in the Mutant Collection

**FIGURE 3:**
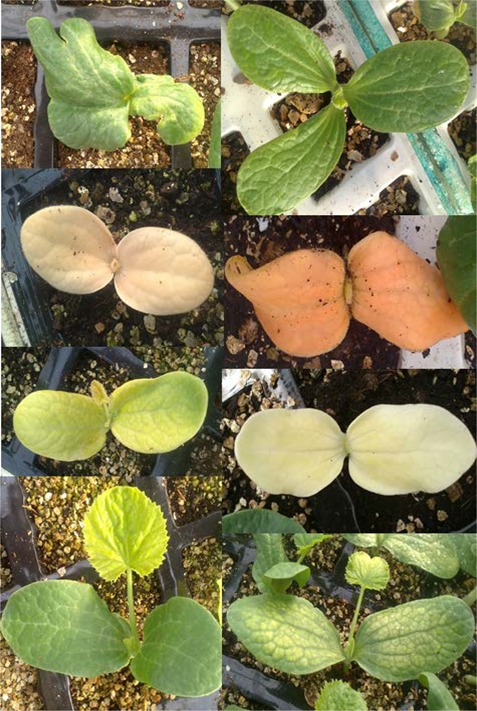
Seedling of *C. pepo* M2 mutant families, showing certain phenotypic alterations: three-cotyledon seedlings, deformation in cotyledons and leaves, variegation and depigmentation in cotyledons and leaves, albino seedlings and dwarfisms.

The quality of the mutant collection was firstly tested by evaluating the phenotypes of 9 plants of each M2 family at the seedling stage of development, focusing on morphological traits that were easily observable in seedlings. The observable changes, which appeared in the cotyledons and leaves of some plants in relation to the genetic background MUC16, were recorded for each M2 family. **Figure 3** shows most of the seedling mutant phenotypes in cotyledons and leaves. A number of M2 families showed alterations in the number and development of cotyledons, other in the development of leaves, and others in the pigmentation of vegetative organs. The percentage of families with typical mutations such as albino plants was 0.54 and 1.2% for the families derived from either 0.2 or 0.3% EMS treatments (**Table 1**). Other mutant phenotypes, including deformations in cotyledons and leaves, were at higher frequency than albinisms, and the frequency of mutant families with depigmentation in cotyledons and leaves was even higher in those derived from the 0.2% EMS treatment, indicating that density of mutations in the plants derived from this weaker treatment is also elevated (**Table 1**). Therefore, at the seedling stage, the total number of phenotypic variations in the mutant library was estimated to be 10.82% (**Table 1**).

**Table 1:** Percentage of phenotypic variations in the seedlings of the 3,751 M2 mutant families of *C. pepo*.

	0.2% EMS (929 families)		0.3% EMS (2,822 families)		Total (3,751 families)
	Number of families	Frequency (%)	Number of families	Frequency (%)	Frequency (%)
Albino	5	0.54	33	1.2	1.04
Tri-cotyledon	13	1.4	29	1.03	1.12
Negative gravitropism	3	0.32	36	1.28	1.04
Cotyledon deformation	10	1.08	95	3.4	2.83
Dwarfism	9	0.97	62	2.2	1.89
Variegation	4	0.43	33	1.2	1.01
Depigmentation	18	1.94	50	1.77	1.81
Orange color	0	0	2	0.07	0.05
Insensitive-ethylene	0	0	4	0.14	0.11
**Total M2 families**	**62**	**6.67%**	**244**	**12.19%**	**10.82%**

**FIGURE 4:**
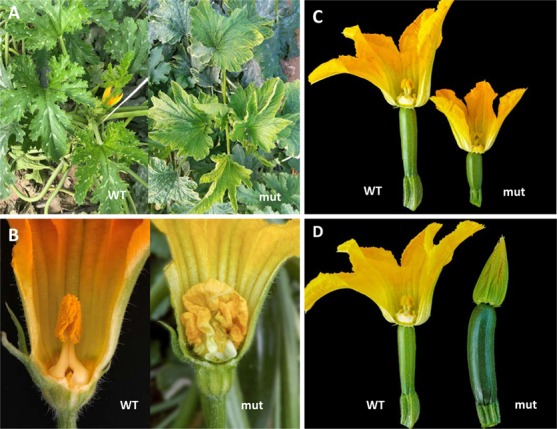
Mutant phenotypes observed in M2 mutant families. **(A)** Alterations in leaf morphology. **(B)** Alterations in male flower development. **(C)** Alterations in female flower size, and ovary shape and size. **(D)** Alterations in fruit set and female flower development (the mutant fruit has a parthenocarpic development).

In adult plants the estimation of phenotypic variations was increased, since we not only phenotyped the vegetative organs of the plant but also flowers and fruits (**Figure 4**). From a total of 284 M2 families, vegetative development alterations were observed in 9.15% of the mutant families (**Table 2**), a similar frequency of vegetative development variations detected in the complete library at the seedling stage. However, in adult plants we also detected different alterations in the morphology of female and male flowers (7.04 and 4.93%, respectively) as well as in fruit color, size and shape (7.04%). The total frequency of phenotypic variations was increased to 28.17% which represents a high frequency of variation for forward genetic approach.

**Table 2:** Percentage of phenotypic variations in adult plants from 284 M2 families derived from the 0.3% EMS treatment.

Phenotypic variation	Number of families	Percentage (%)
Vegetative development	26	9.15
Male flower	14	4.93
Female flowers	20	7.04
Fruit set and development	20	7.04
**Total families affected**	**80**	**28.17%**

### High-Throughput Screening of the Collection for Ethylene Insensitivity

**FIGURE 5:**
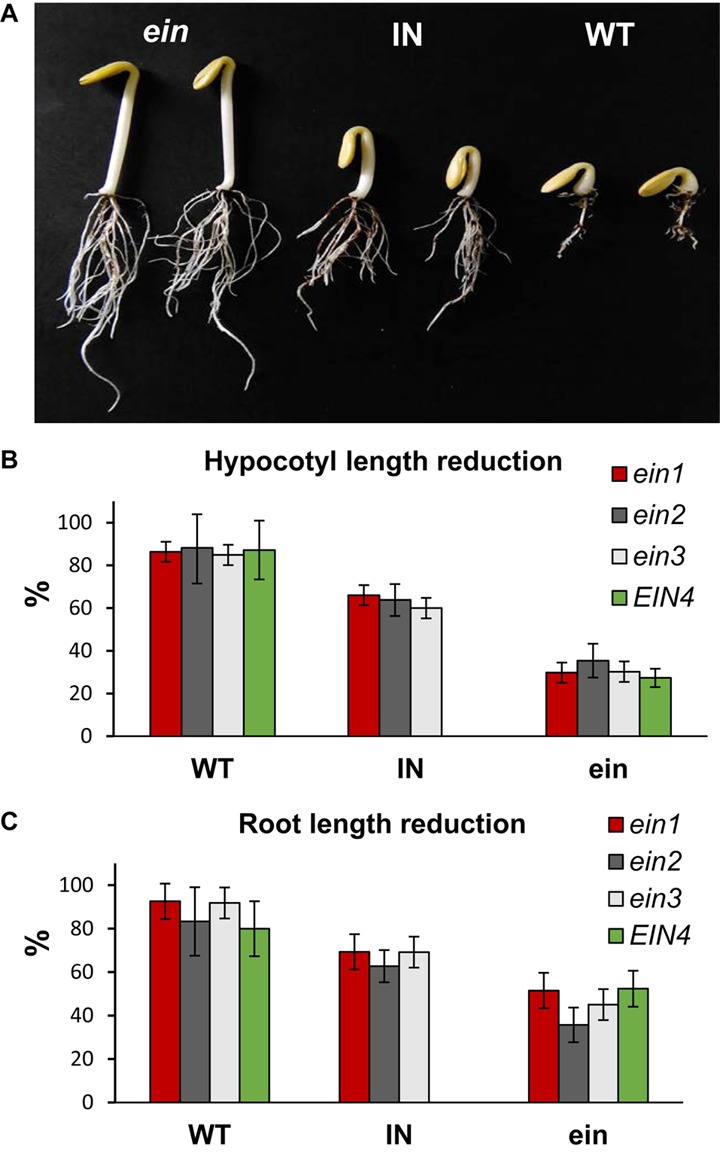
**(A)** Ethylene triple response phenotypes in etiolated seedlings of the *ein1* mutant family of *C. pepo*. WT plants showed a positive triple response, characterized by a reduction in the length of hypocotyl and root, and a thicker hypocotyl. The ethylene insensitive (*ein*) plants do not respond to ethylene and show longer and thinner hypocotyl, and longer roots. Certain plants in the family showed an intermediate (IN) triple response phenotype. **(B,C)** Percentage of reduction of hypocotyl and root length of ethylene treated WT and *ein* mutants in comparison with untreated seedlings of the background genotype MUC16. Errors bars represent standard errors. Note that in the *ein1-3* mutant families there is a distinguishable intermediate (IN) phenotype to ethylene response.

To test the quality of the *C. pepo* mutant library for forward genetic analysis, we performed a high-throughput screening for ethylene insensitivity by using the triple response of etiolated seedlings to ethylene. Nine seeds of each family were germinated and treated with gaseous ethylene for 48 h. In the WT genotypic background (line MUC16) as well as in the vast majority of the M2 families, all plants showed a positive triple response to ethylene. Seedlings displayed a decrease in the length of hypocotyl and roots, an increased hypocotyl thickness and greater curvature of the apical hook, characteristic that were rapidly observed in the germinating seeds (**Figure 5**). Four out of 3,751 families showed a reduced response to ethylene treatment, indicating that some of the plants were insensitive to this hormone. The identified mutants, designated *ein1*, *ein2*, *ein3* and *EIN4*, developed larger and thinner hypocotyls, as well as lager roots in comparison with WT plants of the same family (**Figure 5**). In the *ein1*, *ein2* and *ein3* families, some of plants showed an intermediate phenotype between WT and *ein* mutants.

Only 1–4 plants out of nine analyzed seeds in each mutant family showed an Ein phenotype. To confirm the ethylene insensitivity of these mutant families, we increased the number of plants in segregating progenies of each family and reduced the number of other mutations in each of the families by backcross. The M_2_ mutant plants were backcrossed with the background WT genotype MUC16 for two generations, and the BC_2_ progeny selfed to obtain the BC_2_S_1_ generation. The backcrossed plants had the same Ein phenotype as the original M2 plants. Segregations also demonstrated that the four mutations were semidominant, and that intermediate phenotype for ethylene triple response corresponded to heterozygous plants for the mutations (**Table 3**). Furthermore, by using a higher number of plants for each mutant family, we assessed the reduction in the length of hypocotyls and roots of ethylene treated seeds in comparison with untreated seeds of the background genotype MUC16 (**Figure 5**). In the segregating progenies of *ein1*, *ein2* and *ein3* mutants, we distinguished three phenotypes, although only two phenotypes were found in the *EIN4* family (**Figure 5**).

**Table 3:** Segregation of ethylene insensitive mutants in the backcrossing (BC_1_ and BC_2_), and the first selfed (BC_2_S_1_) generations.

Mutant family	Generation	Number of plants				χ^2^	*p*-value
		WT	INT	*ein*	Expected segregation		
*ein1*	BC_1_	–	62	–		–	–
	BC_2_	20	24	–	1:1	0.36	0.54
	BC_2_S_1_	116	187	115	1:2:1	4.64	0.10
*ein2*	BC_1_	–	79	–		–	–
	BC_2_	42	41	–	1:1	0.01	0.91
	BC_2_S_1_	129	237	119	1:2:1	0.66	0.72
*ein3*	BC_1_	–	42	–		–	–
	BC_2_	31	27	–	1:1	0.28	0.60
	BC_2_S_1_	98	178	86	1:2:1	0.89	0.64
*EIN4*	BC_1_	34	–	30	1:1	0.25	0.62
	BC_2_	120	–	125	1:1	0.10	0.75

*Plants were classified according to their triple response to ethylene: WT, ethylene sensitive seedlings; INT, intermediate response to ethylene; *ein*, ethylene insensitive seedlings. *EIN4* plants only produce male flowers and were unable to be selfed.*

### Density and Spectrum of EMS Mutations

Next generation sequencing technology makes whole-genome sequencing a practical method for mutation identification and mapping. DNA from 40 plants in segregating progenies of two mutant families (L1 and L2) was used for WGS. Since WGS was made simultaneously with the phenotyping of seedlings and plants, these two families were randomly selected from the collection. They were later phenotyped, but no alterations were detected in easily observable morphological traits. Four samples, each one containing a pooled DNA from at least 10 plants in segregating progenies, were sequenced for each family. Sequencing data from the eight samples showed a mean coverage of 92.4 and 95.5% of the whole genome for L1 and L2, respectively. After variant calling for each individual sample, SNVs with GQ > 20 were firstly filtered for a minimum coverage threshold of 8 reads in at least one sample per family, and maximum coverage threshold of 100 reads (median coverage per sample was 35 reads). The regions with higher coverage were filtered out because their high number of SNVs is most likely caused by wrong mapped interspersed DNA sequences. This filtered out 15% of the bases, resulting in a genome coverage of 67 and 67.8% for families L1 and L2, respectively (Supplementary Table 4).

A second filtering step was based on the density of variants, filtering out those SNVs that had another variant closer than 1,000 bp because they were likely caused by sequencing or mapping errors. That reduced the coverage of the reference genome to about 60% in the two families, but also reduced the number of variants to 6,140 in L1 and 5,252 in L2. Most of these variants (4,031) were common to both L1 and L2 (Supplementary Table 4), likely representing spontaneous nucleotide polymorphisms in MUC16 genetic background. They account for 65.7 and 76.8% of SNVs in L1 and L2, respectively. Those that were homozygous in both L1 and L2 (3,136) were probably fixed during the 2–3 extra rounds of selfing that MUC16 reference genotype was subjected before EMS treatments. The other shared SNVs could be spontaneous polymorphisms still segregating in one or the two families. Given that the MUC16 line has more than nine generations of selfing and taking into account that *C. pepo* genome is duplicated (Montero-Pau et al., 2017), we cannot rule out that these shared SNVs were the result of mapping errors in reads coming from duplicated genomic regions.

The specific variants sum a total of 2,209 in L1 and 1,226 in L2 from a total of 144.5 Mb (55.4% of genome) shared by the two families after filtering (Supplementary Table 4). Given the high percentage of spontaneous mutations among SNVs, the homozygous family specific SNVs would mostly be spontaneous polymorphisms that were fixed for the alternative allele in each one of the families after mutagenesis. Therefore, we discarded these family specific homozygous SNVs as truly EMS mutations. The family specific heterozygous SNVs (1,704 and 859 in L1 and L2, respectively) were therefore selected as the tangible EMS induced DNA mutations, which reveals a mutation density of 11.8 mutation/Mb (1 mutation/85 kb) in L1, and 6 mutations/Mb (1 mutation/167 kb) in L2.

**FIGURE 6:**
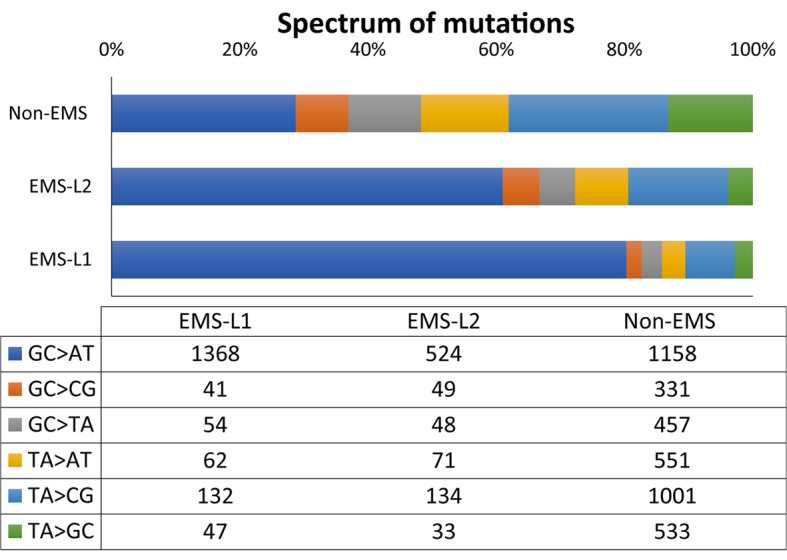
Spectrum and frequency of and EMS and non-EMS mutations in L1 and L2 families. EMS mutations correspond to those that were heterozygous and specific for one of the mutant family. Homozygous family specific SNVs and shared SNVs between L1 and L2 families were considered non-EMS.

The most frequent mutations were the EMS canonical transitions GC to AT (80.3% in L1 and 61% in L2), followed by TA to CG transitions (7 and 15.6%) (**Figure 6**). The frequency of transversion (GC > CG, GC > TA, TA > AT and TA > GC) was relatively low, and summarizes 12.7% in L1 and 23.4% in L2 (**Figure 6**). The transition to transversion ratio was therefore 6.9 and 3.3% in L1 and L2, respectively. The spectrum of mutations in the fraction of SNVs that were discarded as non-EMS (those that were shared by the two families, and those that were homozygous in only one of the families), was not enriched in GC to AT transitions (**Figure 6**), suggesting that they were not likely induced by the mutagen but were polymorphism of the background genotype.

**FIGURE 7:**
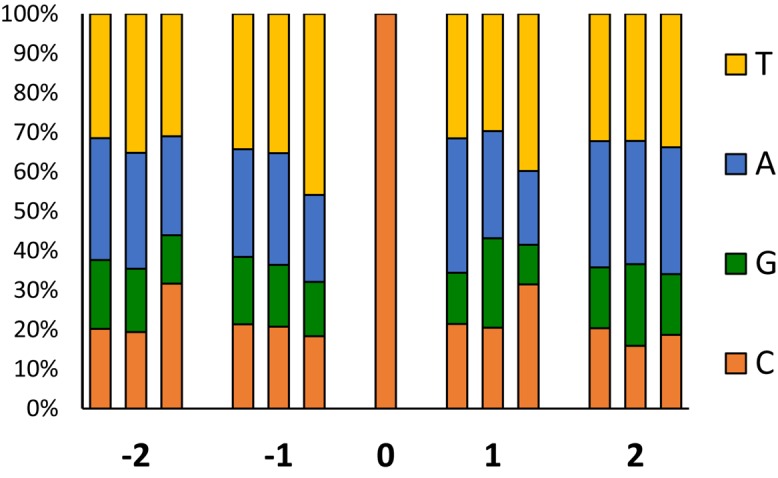
Frequency of the sequences flanking EMS induced mutations. At each position (–2, –1, +1 and +2), left bars correspond to the frequency of bases flanking C in the background genome, the middle bars, to flanking sites of the non-EMS CG > TA variants (natural polymorphisms shared by the two families respect to the reference genome), and the right bars to the canonical EMS mutations (CG > AT).

To investigate sequence biases in EMS-induced mutations, we analyzed the frequency of preferential bases flanking ± 2 positions around the most frequent transitions (GC to AT) respect to the genome background (**Figure 7**). In the background genome, the two bases surrounding a C, were more frequently A and T than C and G. In -1 and +1 positions of the most numerous GC to AT transitions, pyrimidines are more frequent than expected, with significant biases to T in the -1 position and to T and C in the +1 position. No bias was observed in the +2 position, but an excess of Cs occurred at the -2. In comparison with the background genome, these nucleotide biases were not detected around the GC to AT spontaneous SNVs observed in the mutant families (**Figure 7**).

### Distribution and Impact of EMS Mutations

**FIGURE 8:**
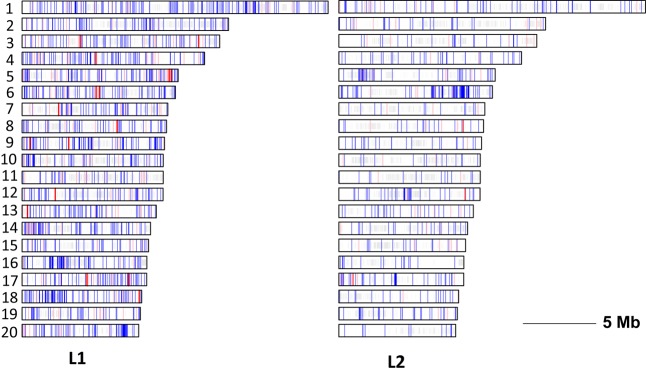
Chromosome distribution (1–20) of EMS-induced mutations in L1 and L2 mutant families. Depending on the predicted impact, EMS-induced variants are plotted as red (high impact), pink (moderate) or blue (low or very low impact). Regions discarded because of the high density of variants are shaded in gray.

The distribution of EMS mutations among the 20 chromosomes of *C. pepo* is shown in **Figure 8**. The mutations were evenly distributed throughout the genome with a density ranging from 5 to 11.7 mutations/Mb in chromosomes 15 and 14 of L1, and 1.5 to 13.6 mutations/Mb on chromosomes 7 and 6 of L2. No preferential distribution was detected among euchromatic and heterochromatic regions in the different chromosomes.

The causative effects of the mutation on gene function was studied by using the SnpEff program. On the base of *C. pepo* genome annotation, many of the EMS mutations were located in regions with low predicted impact on gene function, including intergenic regions (23.6%), downstream and upstream (55%) regions and introns (11.1%). A reduced number of EMS mutations affected, however, to exons (7.3%), 5′- and 3′-UTR regions (2.4%) and splice sites (0.5%), which could likely alter gene function (**Table 4**). Of the 422 exonic mutations 281 (66.6%) were missense mutations, 11 (2.6%) nonsense mutations, and 130 (30.8%) silent (**Table 4**). Regarding their impact, 0.3% of the detected EMS mutations had a predicted high- (e.g., nonsense, frameshift, and splice acceptor or donor mutations), 4.8% a moderate- (e.g., missense mutations), 92.1% a modifier- (e.g., intron and intergenic mutations), and 2.8% a low-impact (e.g., synonymous mutations) on gene function.

**Table 4:** Location and functional impact of EMS mutations in L1 and L2 mutant families of *C. pepo*.

	Mutation	L1	L2	Total	%
Gene region	Exon	311	111	422	7.3
	Intron	479	166	645	11.1
	Splice sites	23	6	29	0.5
	Intergenic	821	548	1,369	23.6
	5′- and 3′- UTR	101	41	142	2.4
	Downstream and upstream	2,202	990	3,192	55
Protein change	Missense	207	74	281	66.6
	Nonsense	9	2	11	2.6
	Silent	95	35	130	30.8
Impact	Low-impact mutations	117	40	157	3.5
	Moderate-impact mutations	207	74	281	6.3
	Modifier-impact mutations	2,776	1,197	3,973	89.7
	High-impact mutations	16	3	19	0.4

*Predicted low-impact mutations correspond to synonymous and splice region variants; Moderate-impact mutations, to missense variants, and high-impact mutations, to stop gained, and splice acceptor and splice donor variants (Supplementary Tables 2, 3).*

Of the 19 high-impact mutations, 10 were nonsense, and 3 and 5 occurred on splice acceptor and splice donor sites of target genes, respectively (**Table 4**). The genes affected by a premature stop codon, which are likely those with higher impact on gene function, code for proteins like: E3 ubiquitin-ligase PRT6 and probable RNA helicase SDE3 in family L2, and for chloroplastic glyceraldehyde-3-phosphate dehydrogenase; multi-bridging factor 1a-like; probable LRR receptor like serine threonine- kinase At4g26540; low-temperature-induced cysteinase; nuclear transcription factor Y subunit B-9; ADP-ribosylation factor-related 1; Protease 2, and aquaporin TIP2-1 in family L1 (Supplementary Tables 2, 3).

## Discussion

### Quality and Usefulness of the *C. pepo* EMS Mutant Library

A mutant platform has been established in the crop species *C. pepo*, consisting of 3,751 M2 families with an average mutation density of 8.9 mutations/Mb (1 mutation per 112 kb). The artificial mutagenesis was found to generate more than 10% of morphological variants in early seedling developmental stages, and more than 30% of morphological alterations in adult plants. This mutation density, detected by both phenomic and genomic approaches, is quite similar or higher to that observed in other plant EMS collections (Dahmani-Mardas et al., 2010; González et al., 2011; Boualem et al., 2014; Vicente-Dólera et al., 2014), indicating that the library will be a high-quality tool for both forward and reverse genetic analyses.

The efficiency of chemical mutagenesis is variable, depending not only on the nature and dosage of the mutagen, but also on the considered species and the background genotype (Chen et al., 2014). The *C. pepo* library was generated with a lower EMS dosage than in other plant species, including the related cucurbit species. *C. melo* collection was generated by 1.5% EMS, while that of *C. sativus* was generated by using 0.5 and 0.75% EMS (Dahmani-Mardas et al., 2010; Boualem et al., 2014). Similar doses (0.7–1.5%) were used in tomato (Minoia et al., 2010), maize (Till et al., 2004) and rice (Till et al., 2007). Despite the use of lower EMS dosage, the *C. pepo* library did not reduce the mutation density, suggesting that *C. pepo* is more sensitive to EMS than other species. Seed germination was similarly affected in the different species, but the more harmful effect of EMS treatment in squash was observed on plant fertility. Consequently, the selection of EMS dose could not be made on the basis of seed germination but based on M1 plant fertility. At the end an EMS dosage was utilized (0.2 and 0.3%) that resulted in 100% of M1 seed survival, but reduced fertility to about 50%, and allowed therefore the production of large populations of viable M2 seeds.

The alterations found in seedlings morphological traits, which is a good indicator of a high-quality mutant collection (Wang et al., 2008), reached 10.82%, is similar to that reported for other well characterized mutant collections. Thus, the percentage of M2 families with albino and chlorophyll deficient families in our collection (1.85%) was very similar to the 1.3% rate found in a previous squash library (Vicente-Dólera et al., 2014), as well as the 0.6 and 2.1% rates observed in the mutant collection of *C. sativus* (Boualem et al., 2014), and muskmelon (González et al., 2011), respectively. Similar frequency of albino mutants, were found in tomato (1.7%; Minoia et al., 2010) and in common bean (1.53%; Porch et al., 2009). The frequency of the dwarf phenotype in our library (1.89%) was also similar to that observed in other species, including tomato, squash and soybean (Minoia et al., 2010; Vicente-Dólera et al., 2014; Tsuda et al., 2015). These data confirm the efficiency of the mutagenesis and demonstrate that the generated mutant library has a high density of mutations, where potential mutant phenotypes can be certainly found by a forward genetic approach.

The frequency of mutant phenotypes in adult plants (28.17%) further encourages the use of our library in forward genetic analyses. Many of them were variations in vegetative developmental traits such as leave shape and color, and growth habit, but many of the mutant families were altered in male and female flower development (11.97%), including sex determination and sex expression mechanisms (**Table 2**). The high-throughput screening for ethylene insensitivity by using the triple response validate the use of this mutant library in forward genetic approach. A high throughput phenomic screening with more than 35,000 plants resulted in four Ein mutants that showed a reduced response to ethylene in root length and hypocotyl length and thickness. This hormone controls flower and fruit development (Martínez et al., 2013), sexual expression and sex determination (Manzano et al., 2010, 2011, 2013), and chilling injury in refrigerated zucchini fruit (Megías et al., 2016). Therefore, these mutants could be of value not only for investigating the function of ethylene receptor and signaling genes in *C. pepo*, but also for breeding a number of agronomic traits in this and other *Cucurbita* crop species, providing they can be crossed with *C. pepo*. Moreover, knowledge of gene function in *C. pepo* would permit the biotechnological manipulation of other plant species through transgenesis or genome-editing technologies.

### Density, Spectrum, Distribution and Impact of EMS Mutations

The mutation density in the squash mutant platform, estimated by WGS of two independent mutant families, was high if compared with other libraries. In addition to sequence quality and coverage, we also filtered SNVs that likely resulted from sequencing and mapping errors (regions with excessive coverage and with high mutation density, probably caused by wrong mapping of repetitive DNA reads), and spontaneous point mutations (SNVs shared by the two families). Discarding clustered SNVs (those that were separated less than 1000 bp) is also in accordance with a study in *Drosophila*, where it was determined that clustered SNPs, which in this case were less than 500 bp apart from one another, were actually natural polymorphisms that arose from gene conversion events (Blumenstiel et al., 2009). After filtering, a total of 2,563 EMS mutations were identified in both L1 and L2 families. The average density was estimated as 1 mutation/111 kb in 144.5 Mb of scored genome, which is slightly higher respect to that so far available and published for a smaller squash mutant population of 768 M2/M3 families (1/133 kb; Vicente-Dólera et al., 2014). The estimated mutation density was also higher in comparison to that published for other EMS mutant collections in the related cucurbit species melon (1 mutation/573 kb; Dahmani-Mardas et al., 2010) and cucumber (1 mutation/1147 kb; Boualem et al., 2014), although in all these cases, the estimations were based on the TILLING of specific target genes and not on WGS. The higher mutation density observed in squash in comparison with other EMS families in related cucurbit species, could be related with the whole genome duplication event observed in *Cucurbita* species (Montero-Pau et al., 2017), which could help in withstanding the mutagen action, as appears to occur in tetraploid and hexaploid wheat, where observed EMS mutation density reaches 1/40 and 1/24 kb, respectively (Slade et al., 2005). The twofold differences in mutation density observed between the two sequenced families has been also reported in mutant libraries of other species like soybean (Tsuda et al., 2015), tomato (Shirasawa et al., 2016), and *Caenorhabditis elegans* (Sarin et al., 2010), where differences among certain families reached up to tenfold.

The most frequent EMS-induced mutations found in the mutant collection were GC to AT transitions, which are known to result from the alkylation of guanine residues. In other mutant libraries of *Arabidopsis*, maize and pea, the frequency of GC to AT transitions represent more than 99% of the EMS mutations (Greene et al., 2003; Till et al., 2004; Dalmais et al., 2008). Whole genome re-sequencing allowed the identification of 80.3 and 61% of GC > AT transitions in L1 and L2, respectively, but other less frequent transitions (TA > CG) and transversions (TA > AT, GC > CG, TA > GC and GC > TA) were also identified in the EMS-induced fraction. Although one could think that these non-canonical mutations are false positives, their occurrence and proportion is consistent with that found in other studies in *Drosophila* (Cooper et al., 2008; Blumenstiel et al., 2009), tomato (Minoia et al., 2010; Shirasawa et al., 2016), soybean (Cooper et al., 2008), rice (Till et al., 2007) and barley (Caldwell et al., 2004), suggesting that, although caused by unknown mechanisms, they are also produced by EMS.

The spectrum of spontaneous mutations occurring in the genome of *C. pepo* was different to that generated by EMS. In this fraction, identified as SNVs shared by the two families under analysis or homozygous for one of the families, the percentage of transitions GC to AT (28.6%) and TA to CG (24.8%) was very similar, while the percentage of transversions reached 46.4%. Moreover, the transition to transversion ratio of spontaneous mutations (1.15) is very different to that in the EMS-induced mutations (6.9 and 3.3 in L1 and L2, respectively), which also strengthen the filtering process followed for the selection of EMS-induced mutations in our study.

The artificial mutations were evenly dispersed throughout the 20 chromosomes of *C. pepo*, and occur randomly in the genome, independently of their location in euchromatic or heterocromatic regions. However, EMS action does not appear to be randomly occurring but is prone to happen in some specific DNA sequences. We found that the two contiguous bases at both sides of the base substitution GC to AT transitions were biased respect to the genome background, with an excess of pyrimidine at -1 and +1 positions, and an excess of C at the -2 position (**Figure 7**). These data are in accordance to that found in other plant species, including tomato (Shirasawa et al., 2016) and *Arabidopsis* (Greene et al., 2003).

## Conclusion

A large mutation platform has been developed in *C. pepo*, containing 3,751 independent families with an average mutation frequency of 1/111 kb. The mutations are evenly distributed along the complete genome and consist of more than 75% of GC to AT transitions, which are the canonical mutations generated by EMS. The putative impact of the mutation detected by WGS, and the phenotypic variation observed in the new squash-mutant platform corroborate its usefulness in high-throughput mutation discovery for both gene function analysis and plant breeding. The high-throughput screening of the whole library by using the triple response to ethylene has yielded a total of 4 Ein mutants that are presently being analyzed, and other screenings for biotic and abiotic stresses are underway with the aim to detect novel alleles and new gene functions.

## Author Contributions

AG conducted most of the experiments. EA collaborated in phenotyping and figures development. SM, CM, and ZM generated the EMS mutant population. GC and JR collaborated in the phenotyping of M2 adult plants. GP and SB performed the bioinformatic analysis and drafted the sequencing methods. MJ and AG interpreted the mutation detection data and drafted the manuscript. DG revised the manuscript draft. MJ coordinated the study and experiments designing.

## Conflict of Interest Statement

The authors declare that the research was conducted in the absence of any commercial or financial relationships that could be construed as a potential conflict of interest.

## Footnotes

1 cucurbitgenomics.org

2 cucurbitgenomics.org

## Abbreviations

Ein: ethylene insensitive
EMS: ethyl methanosulfonate
L1, L2: mutant family 1 and mutant family 2
NGS: next generation sequencing
SNP: single nucleotide polymorphism
SNV: single nucleotide variant
TILLING: Targeting-induced local lesions in genomes
WGS: whole genome sequencing
