# Supplementary material for: Phenomic and Genomic Characterization of a Mutant Platform in Cucurbita pepo
Source: Front Plant Sci. 2018 Aug 3;9:1049. doi: 10.3389/fpls.2018.01049 (PMC6085476; doi:10.3389/fpls.2018.01049)
Supplement: Supplementary file 1 [file Table_1.DOCX]

Supplementary Material

Phenomic and genomic characterization of a mutant platform in *Cucurbita pepo*

Alicia García^1^, Encarnación Aguado^1^, Genis Parra^2^, Susana Manzano^1^, Cecilia Martínez^1^, Zoraida Megías^1^, Gustavo Cebrián^1^, Jonathan Romero^1^, Sergi Beltrán^2^, Dolores Garrido^3^, Manuel Jamilena^1*^

***Correspondence:** Dr. Manuel Jamilena: [mjamille@ual.es](mailto:mjamille@ual.es)

| **Supplementary Table 1. Sequence and coverage statistics. Number of sequenced, unmapped and duplicate reads. Mean and median coverage and percent of genomic bases with a fold coverage higher than 5.** | | | | | | | | |
| --- | --- | --- | --- | --- | --- | --- | --- | --- |
| **Sample** | **Seq. reads** | **Unmap reads** | **% Unmap** | **Dup. reads** | **% dup** | **Mean coverage** | **Median coverage** | **% Coverage**  **>5x** |
| **L1.1** | 17,470,139 | 1,189,573 | 6.8 | 1,289,252 | 7.37 | 16.37 | 9 | 82.1 |
| **L1.2** | 17,472,279 | 1,100,148 | 6.29 | 1,272,279 | 7.28 | 16.38 | 10 | 82.6 |
| **L1.3** | 15,557,026 | 954,025 | 6.13 | 1,054,410 | 6.77 | 14.64 | 9 | 78.9 |
| **L1.4** | 17,434,357 | 1354914 | 7.77 | 1,278,591 | 7.33 | 16.33 | 9 | 82.2 |
| **L2.1** | 18,583,646 | 1253795 | 6.74 | 1,410,944 | 7.59 | 17.39 | 10 | 83.7 |
| **L2.2** | 18,245,737 | 1,055,990 | 5.78 | 1,373,955 | 7.53 | 17.08 | 10 | 82.9 |
| **L2.3** | 19,185,343 | 1,046,213 | 5.45 | 1,557,843 | 8.11 | 17.9 | 10 | 83.8 |
| **L2.4** | 13,983,971 | 870,083 | 6.22 | 903,141 | 6.45 | 13.18 | 8 | 73 |
