# Supplementary material for: Phenomic and Genomic Characterization of a Mutant Platform in Cucurbita pepo
Source: Front Plant Sci. 2018 Aug 3;9:1049. doi: 10.3389/fpls.2018.01049 (PMC6085476; doi:10.3389/fpls.2018.01049)
Supplement: Supplementary file 2 [file Table_2.XLSX]

Supplementary Material

Phenomic and genomic characterization of a mutant platform in *Cucurbita pepo*

Alicia García^1^, Encarnación Aguado^1^, Genis Parra^2^, Susana Manzano^1^, Cecilia Martínez^1^, Zoraida Megías^1^, Gustavo Cebrián^1^, Jonathan Romero^1^, Sergi Beltrán^2^, Dolores Garrido^3^, Manuel Jamilena^1*^

***Correspondence:** Dr. Manuel Jamilena: mjamille@ual.es

| **Supplementary Table 4.** Filtering steps of the original sequencing data of L1 and L2 mutant families to discard false positive mutations induced by EMS | | | | | | | | | |  |
| --- | --- | --- | --- | --- | --- | --- | --- | --- | --- | --- |
|  |  | **L1** |  |  | **L2** | |  | **L1 + L2** | | |
|  |  | **Base coverage**  **(% Ref. genome)** | **No. SNVs** |  | **Base coverage**  **(% Ref. genome)** | **No. SNVs** |  | **Shared bases**  **(L1 and L2)** | **Shared SNVs**  **(L1 and L2)** | |
| BQ>20 |  | 198,385,685  (76.1 %) |  |  | 198451381  (76.1%) |  |  |  |  | |
| Filtering out bases with coverage lower than 8 and higher than 100 |  | 174752501  (67%) | 190,333 |  | 176805948  (67.8%) | 183,167 |  | 162504354 (62.3%) |  | |
|  |  |  |  |  |  |  |  |  |  |  |
| Filtering regions with excess of variants: more than 1 SNV/kb |  | 157579060  (60.4%) | 6,140 |  | 160330390  (61.5%) | 5,257 |  | 144477323 (55.4%) | 4,031 | |
|  |  |  |  |  |  |  |  |  |  |  |
| Filtering out common SVNs in shared bases between L1 and L2 |  | 144477323  (55.4%) | 2,109 |  | 144477323  (55.4%) | 1,226 |  |  |  | |
|  |  |  |  |  |  |  |  |  |  |  |
| Homozygous SVNs |  |  | 405 |  |  | 367 |  |  |  | |
| **Heterozygous SVNs:**  **EMS mutations** |  |  | **1704 (11.8/Mb)** |  |  | **859 (6/Mb)** |  |  |  |  |
